# Dorsal root ganglion neurons regulate the transcriptional and translational programs of osteoblast differentiation in a microfluidic platform

**DOI:** 10.1038/s41419-017-0034-3

**Published:** 2017-12-13

**Authors:** Diana Isabel Silva, Bruno Paiva dos Santos, Jacques Leng, Hugo Oliveira, Joëlle Amédée

**Affiliations:** 1 Tissue Bioengineering, University of Bordeaux, U1026, 33076 Bordeaux, France; 2 Tissue Bioengineering, INSERM, U1026, 33076 Bordeaux, France; 3 University of Bordeaux, LOF, UMR5258, 33600 Pessac, France; 4 CNRS, LOF, UMR5258, 33600 Pessac, France; 5 Solvay, LOF, UMR5258, 33600 Pessac, France

## Abstract

Innervation by the sensory nervous system plays a key role in skeletal development and in orchestration of bone remodeling and regeneration. However, it is unclear how and in which bone cells can sensory nerves act to control these processes. Here, we show a microfluidic coculture system comprising dorsal root ganglion (DRG) neurons and mesenchymal stem cells (MSCs) that more faithfully represents the *in vivo* scenario of bone sensory innervation. We report that DRG neurons promote the osteogenic differentiation capacity of MSCs, by mediating the increase of alkaline phosphatase activity and the upregulation of osteoblast-specific genes. Furthermore, we show that DRG neurons have a positive impact on Cx43 levels in MSCs during osteoblastogenesis, especially at an early stage of this process. Conversely, we described a negative impact of DRG neurons on MSCs N-cadherin expression at a later stage. Finally, we demonstrate a cytoplasmic accumulation of β-catenin translocation into the nucleus, and subsequently Lymphoid Enhancer Binding Factor 1—responsive transcriptional activation of downstream genes in cocultured MSCs. Together, our study provides a robust body of evidence that the direct interaction of DRG neurons with MSCs in a bone-like microenvironment leads to an enhancement of osteoblast differentiation potential of MSCs. The osteogenic effect of DRG neurons on MSCs is mediated through the regulation of Cx43 and N-cadherin expression and activation of the canonical/β-catenin Wnt signaling pathway.

## Introduction

Specific bone cell activity, local factors, and systemic hormones regulate the skeletal morphogenesis and homeostasis^1^. However, accumulated evidence has demonstrated that central and peripheral nervous systems play also an essential role in bone formation, remodeling, and regeneration^2^. Histologic studies revealed the presence of sensory and sympathetic nerve fibers, frequently accompany with blood vessels, in the periosteum, trabecular and cortical bone, bone marrow, and epiphyseal plate^3,4^. Interestingly, the distribution pattern of nerve fibers was shown to vary according to the bone region. The periosteum and mineralized bone regions with high osteogenic activity present an intensive network of sensory and sympathetic nerve fibers expressing neurotransmitters and neurotrophins^3–8^. Additionally, bone cells have shown to actively respond to a wide range of neuronal signaling molecules through specific receptors^9^. A large number of animal experiments has reinforced these observations by demonstrating that denervation has a detrimental effect on bone development, homeostasis and repair^10,11^. More recently, a work published by Fukuda and coleagues^12^ revealed that sensory nervous system (SNS) is the main regulator of bone formation and remodeling in mice. In this work, the authors identified the Semaphorin 3A (Sema3A) produced in sensory neurons as a crucial protein for proper bone mass accrual. Similarly, it was reported that intravenous administration of Sema3A in Sema3A^−/−^ mice prevents bone loss^13^.

Taken together, these *in vivo* findings have proven that SNS plays an extensive and pivotal role in bone physiology and pathophysiology. However, this evidence at molecular/cellular levels is poorly understood. Indeed, the underlying molecular mechanisms and bone cells under direct control of SNS remain unclear and further studies are required. Additionally, the majority of *in vitro* studies comprising sensory neurons and bone cells were performed with conventional cocultures that are far from mimicking the *in vivo* scenario of bone sensory innervation^14–16^.

In this study, we used a microfluidic coculture system in order to explore in a more accurate microenvironment the effect of dorsal root ganglion (DRG) neurons on the ability of MSCs to undergo osteoblast differentiation. Our results demonstrate that DRG neurons enhance the osteoblastogenesis by regulating the canonical/β-catenin Wnt signaling pathway and expression of osteoblast-related genes/proteins in MSCs.

## Results

### Microfluidic devices allow the neurite outgrowth within the MSCs compartment

**Fig. 1:**
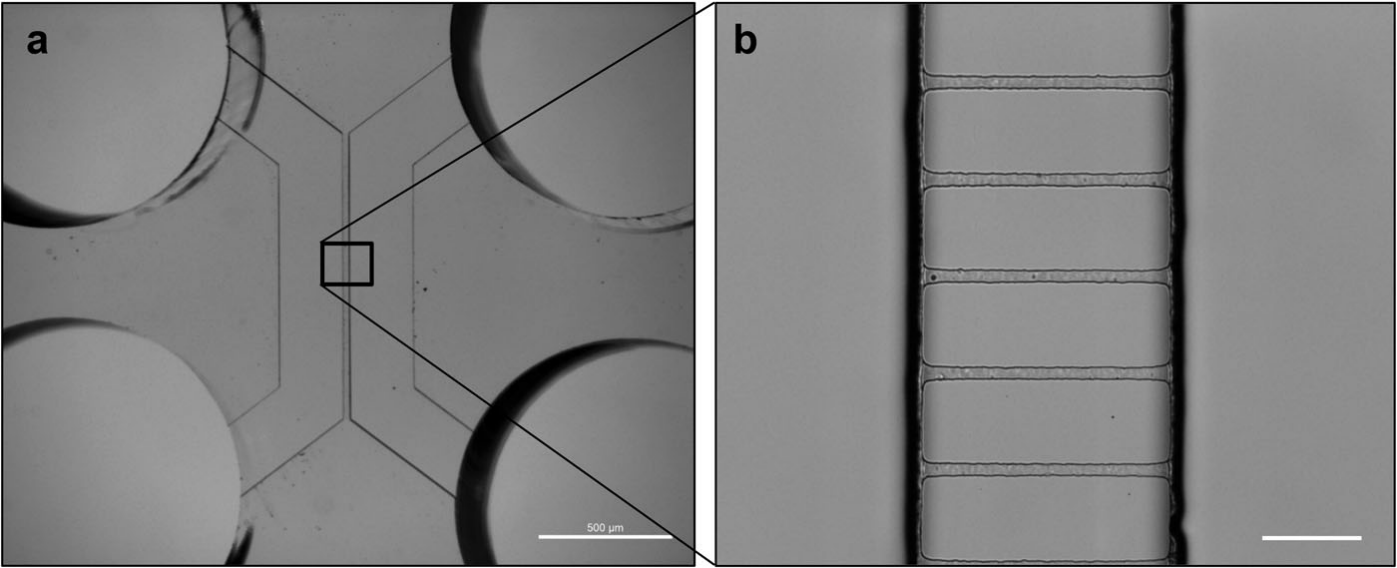
Microfluidic coculture device Conventional photolithography and soft lithography techniques were used to create the microfluidic device. Phase contrast microphotograph of the **a** device and **b** microgrooves with the following dimensions: 3.6 µm of high, 7 µm wide, and 148 µm long (spaced 48 µm one another). Scale bar = 10 µm

The identification of the role of the SNS in the regulation of osteogenesis raises the fundamental question whether DRG neurons operate directly in osteoblast differentiation of MSCs. To explore this hypothesis, we fabricated a microfluidic platform, whose design was adapted from previously validated geometric patterns for neuronal studies^17^. Conventional lithography techniques were used to create the microfluidic device, which consist of a miniaturized two-chamber system (Fig. 1a) connected by well-defined microgrooves (Fig. 1b). This microdevice enables the  separate culture of the cell bodies of DRG neurons and the target cells, using specific culture media for each cell type,  and assess the distinct gene/protein profile of each cell population^18,19^.

Primary cultures of DRG neurons and MSCs were obtained from adult Wistar rats (Supplementary Figure S1). The morphology of DRG neurons was analyzed by immunofluorescence (IF) for calcitonin gene-related peptide (CGRP) (Supplementary Figure S1A). The spindle-shaped morphology of bone marrow MSCs was verified under an optical microscope (Supplementary Figure S1B), and the expression of positive and negative cell surface markers was confirmed by flow cytometry (Supplementary Figure S1C).

**Fig. 2:**
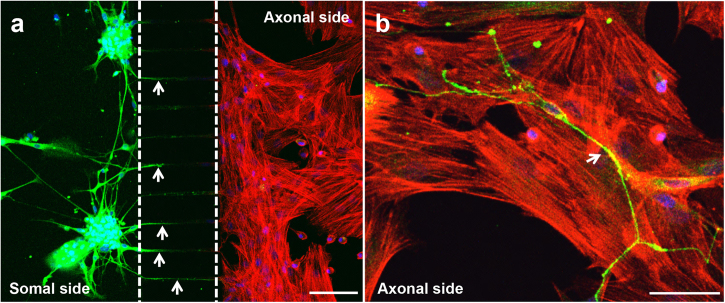
Neurites cross the microgrooves to the axonal side of the microfluidic device Sensory neurons derived from rat DRG (5 × 10^4^ cells/cm^2^) and rat bone marrow MSCs (10^4^ cells/cm^2^) were cocultured in microfluidic devices for 7 days. DRG neurons were maintained in DMEM supplemented with 2% (v/v) B-27 and 1 μM AraC; MSCs were incubated in OIM composed of DMEM-low glucose with 10% (v/v) FBS, 1 × 10^−9^ M dexamethasone, 10 mM β-glycerophosphate, and 50 μg/mL ascorbic acid. (**a** and **b**) The presence of neurites reaching MSCs was evaluated on day 7 of coculture by IF using an antibody directed against a neuronal specific marker (β-III Tubulin) coupled to Alexa Fluor^®^ 488 (green), and DAPI (nuclei; blue) under a confocal microscope. Actin filaments of MSCs were stained using Alexa Fluor^®^ 568 (red)-conjugated phalloidin. Arrows point to neurites. Scale bar = 100 µm **a**, 50 µm **b**

Prior to assembling the coculture system in the microfluidic device (Fig. 2), MSCs were cultured alone under standard conditions until they attained confluence. Thereafter, DRG neurons were seeded into the somal side of the microdevice, while MSCs were plated into the axonal side (Fig. 2a). DRG neurons were incubated in growing medium and MSCs were cultured with osteogenic induction medium (OIM). IF against β-III Tubulin was performed on day 7 to observe neurite outgrowth within the microfluidic platform. We observed that DRG neurons spread neurites from the neural cell bodies, placed on the somal side, toward the MSCs compartment through microgrooves (Fig. 2a, arrows). Notably, neurites could be detected within the axonal side in close proximity with MSCs from day 4 of coculture (Fig. 2b, arrow).

**Fig. 3:**
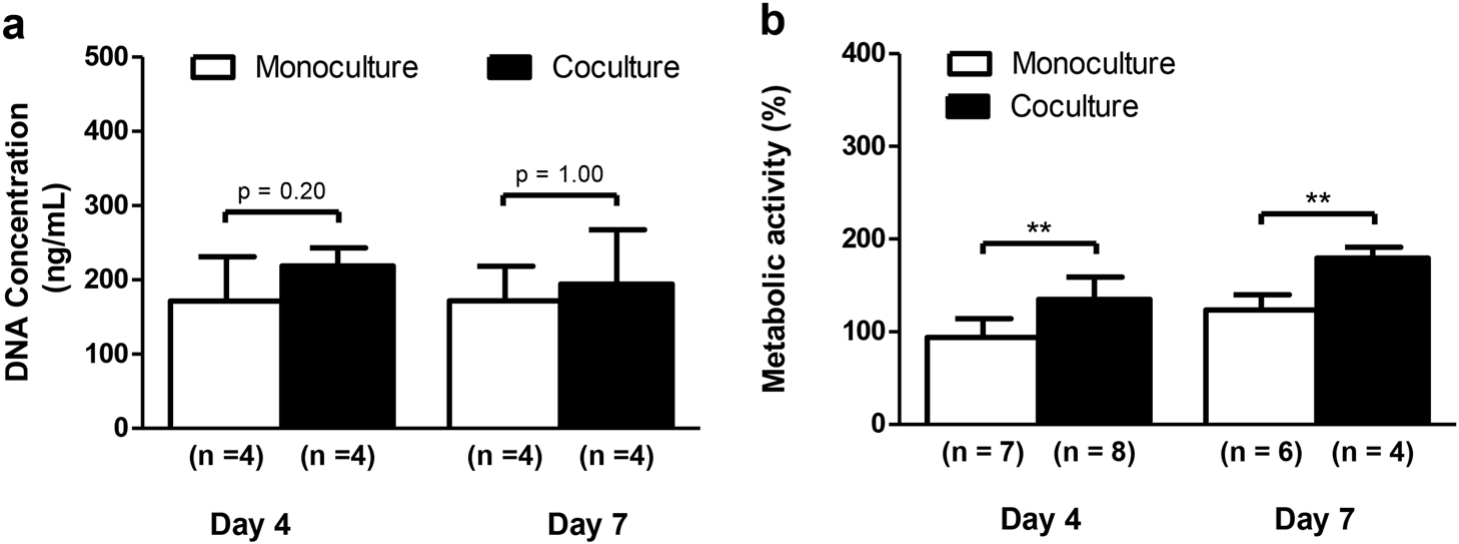
DRG neurons stimulate the metabolic activity in MSCs Sensory neurons derived from rat DRG (5 × 10^4^ cells/cm^2^) and rat bone marrow MSCs (10^4^ cells/cm^2^) were cocultured in microfluidic devices for 7 days. DRG neurons were maintained in DMEM supplemented with 2% (v/v) B-27 and 1 μM AraC; MSCs were incubated in OIM composed of DMEM-low glucose with 10% (v/v) FBS, 1 × 10^−9^ M dexamethasone, 10 mM β-glycerophosphate, and 50 μg/mL ascorbic acid. **a** The DNA concentration of MSCs was determined at 4 and 7 days of coculture by CyQUANT™ Cell Proliferation Assay. **b** The relative metabolic activity of MSCs was measured at 4 and 7 days of coculture by resazurin-based assay and normalized to the monoculture levels on day 4. Data expressed as mean ± SD. (*n*) indicates the total number of samples for each group. **p < 0.01 statistically different from monoculture. The results represent three independent experiments.

To assess the impact of coculture with DRG neurons on MSCs proliferation in the microfluidic platform, we evaluated DNA content and metabolic activity in MSCs after 4 and 7 days of coculture in the presence of OIM (Fig. 3). No differences were found in the DNA concentration between MSCs in mono and coculture (Fig. 3a). However, we observed an increase of 40% on day 4 and more than 55% on day 7 in the metabolic activity of cocultured MSCs in relation to their respective control MSCs (Fig. 3b). These results indicate that coculture with DRG neurons stimulates the metabolic activity of MSCs during osteoblastogenesis.

### DRG neurons enhance the differentiation of MSCs towards the osteoblast lineage

**Fig. 4:**
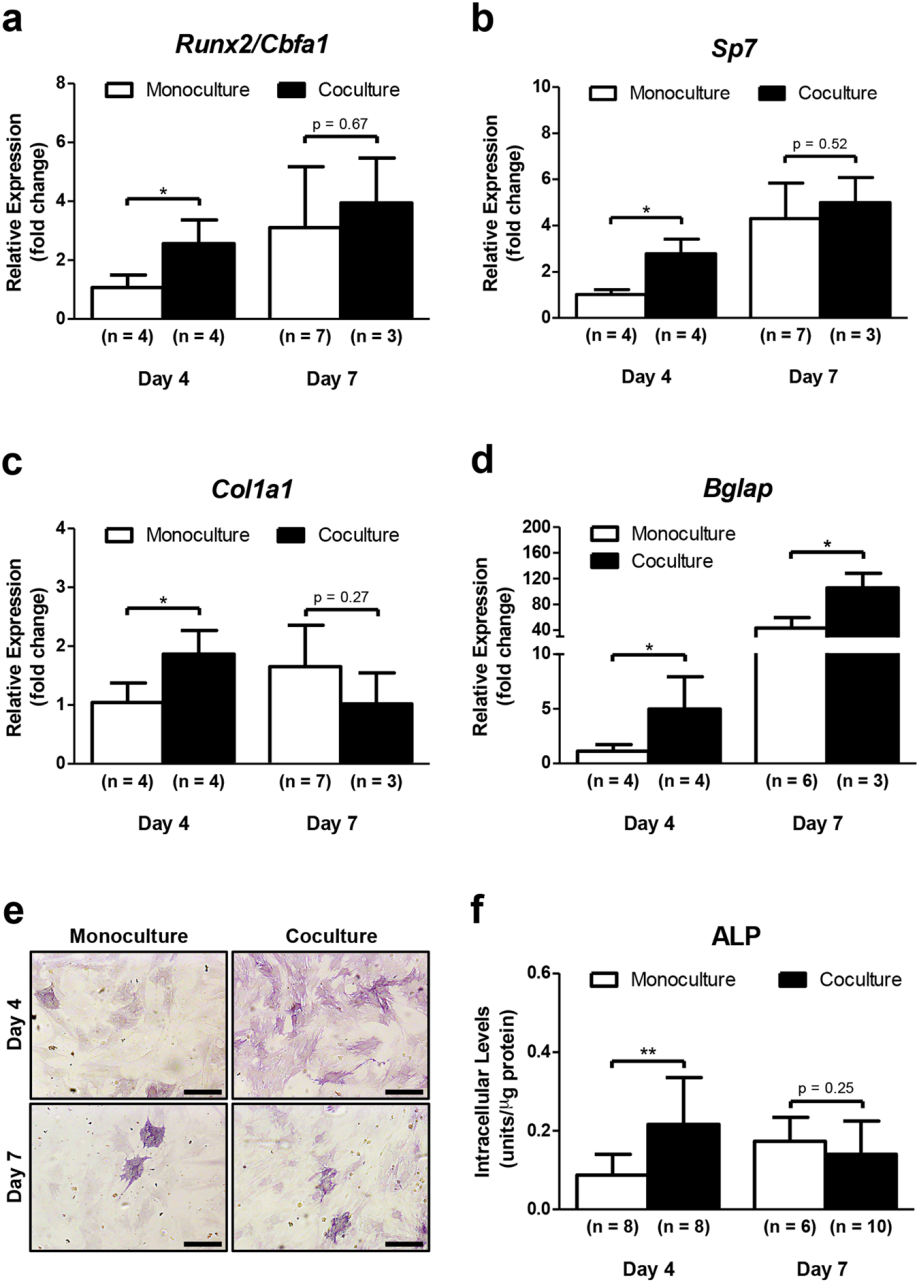
DRG neurons enhance the osteoblast differentiation ability of MSCs Sensory neurons derived from rat DRG (5 × 10^4^ cells/cm^2^) and rat bone marrow MSCs (10^4^ cells/cm^2^) were cocultured in microfluidic devices for 7 days. DRG neurons were maintained in DMEM supplemented with 2% (v/v) B-27 and 1 μM AraC; MSCs were incubated in OIM composed of DMEM-low glucose with 10% (v/v) FBS, 1 × 10^−9^ M dexamethasone, 10 mM β-glycerophosphate, and 50 μg/mL ascorbic acid. **a–d** Expression profile of *Runx2*, *Sp7*, *Col1a1*, and *Bglap* in MSCs was assessed at 4 and 7 days of coculture by RT-qPCR and depicted as a relative ratio to the housekeeping gene *Hprt1* normalized to the monoculture levels on day 4. **e** and **f** Alp activity in MSCs was analyzed at 4 and 7 days of coculture by Alp activity quantification assay and cytochemical staining. Scale bar = 300 µm. Data expressed as mean ± SD. (*n*) indicates the total number of samples for each group. *p < 0.05; **p < 0.01 statistically different from monoculture. The results represent three independent experiments.

The osteoblast formation from MSCs involves several cellular/molecular processes, including the transcriptional regulation of bone phenotyping genes^20^. To investigate whether the coculture with DRG neurons had an influence on the osteoblast differentiation capacity of MSCs in the microdevice, we analyzed the transcriptional expression of osteoblast-marker genes in MSCs by RT-qPCR after 4 and 7 days of coculture in OIM (Fig. 4a–d). Genes of interest included runt-related transcription factor 2 (*Runx2*), also known as core-binding factor subunit alpha-1 (Cbfa1), specificity protein transcription factor 7 (*Sp7*, encoding osterix), collagen type I alpha 1 chain (*Col1a1*), and bone gamma-carboxyglutamate protein (*Bglap*, encoding osteocalcin). *Runx2/Cbfa1* and *Sp7* are two master regulatory genes required for osteoblast differentiation^21–24^. Runx2/Cbfa1 is the earliest and the most specific transcription factor of osteoblastogenesis, guiding the immature bone development. This protein can regulate the expression of important osteoblastic genes, regulate *Runx2* itself, and also interacts with other proteins expressed in osteoblasts, including osterix, to modulate the osteoblast genetic program^25–27^. *Col1a1* is considered an early marker for osteoblast differentiation associated to bone ECM formation^28,29^. On the other hand, *Bglap* is characterized as a highly specific late osteoblastic marker related to bone ECM mineralization^30^. In our research, we found a significant upregulation of *Runx2/Cbfa1* (2.5-fold change), *Sp7* (2.8-fold change), *Col1a1* (2-fold change), and *Bglap* (3.4-fold change) in MSCs cocultured with DRG neurons for 4 days, when compared with MSCs in monoculture (Fig. 4a–c). Similarly, an increase of *Bglap* expression was detected on day 7 in MSCs in coculture (3.4-fold change compared with monoculture at the same timepoint) (Fig. 4d). Interestingly, there were no differences in the transcription of osteoblast-related genes between mono and cocultured MSCs under standard culture conditions without osteogenic stimulation (Supplementary Figure S2A-E). These findings reveal that coculture with DRG neurons leads to an increase in the transcriptional expression of osteoblast-specific genes in MSCs during osteoblastogenesis.

Alkaline phosphatase (Alp) is another early marker for osteoblast lineage development participating in the bone ECM deposition. Intracellular Alp quantification assay and cytochemical staining were conducted to further understand the effect of coculture with DRG neurons on osteoblast differentiation capacity of MSCs (Fig. 4e, f). Accordingly, MSCs cocultured with DRG neurons for 4 days exhibited more intense purple staining for Alp than monocultured MSCs (Fig. 4e). On the other hand, there were no differences between MSCs in mono and coculture after 7 days, showing both for cell conditions a weak Alp-staining (Fig. 4e). Identical results were achieved with Alp activity quantification assay (Fig. 4f). Additionally, no differences were found in the Alp activity between mono and cocultured MSCs under standard culture conditions (Supplementary Figure S2F). These results suggest that coculture with DRGs neurons promotes the osteogenic activity in MSCs during osteoblast differentiation.

Taken together, these results strongly suggest a direct involvement of SNS in the regulation of osteoblastogenesis in MSCs.

### DRG neurons modulate the expression of Cx43 and N-cadherin in MSCs during osteoblastogenesis

**Fig. 5:**
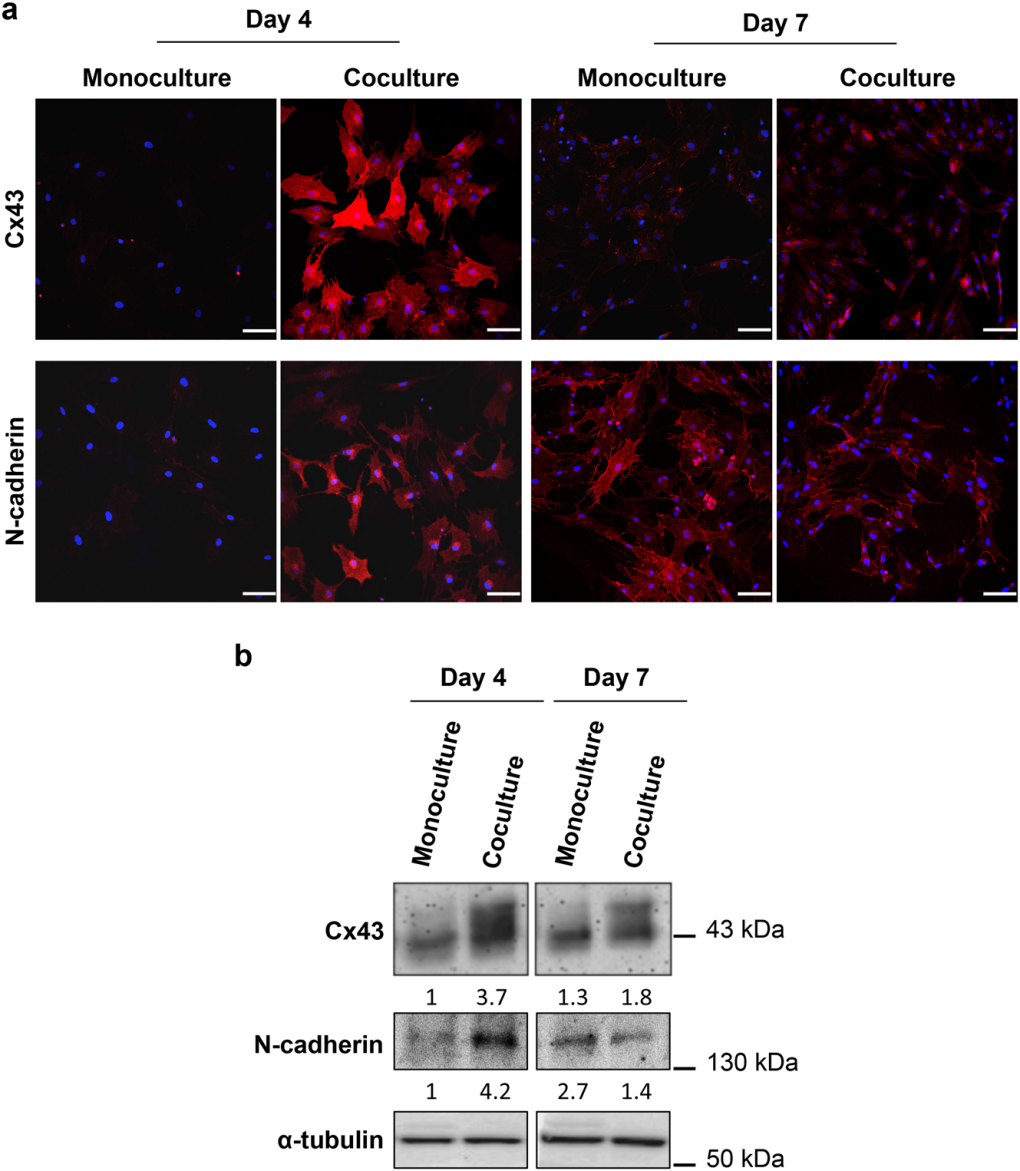
DRG neurons have an impact on Cx43 and N-cadherin expression in MSCs during osteoblast differentiation Sensory neurons derived from rat DRG (5 × 10^4^ cells/cm^2^) and rat bone marrow MSCs (10^4^ cells/cm^2^) were cocultured in microfluidic devices for 7 days. DRG neurons were maintained in DMEM supplemented with 2% (v/v) B-27 and 1 μM AraC; MSCs were incubated in OIM composed of DMEM-low glucose with 10% (v/v) FBS, 1 × 10^−9^ M dexamethasone, 10 mM β-glycerophosphate, and 50 μg/mL ascorbic acid. **a** Subcellular distribution of Cx43 and N-cadherin in MSCs was evaluated at 4 and 7 days of coculture by IF using antibodies directed against Cx43 and N-cadherin coupled to Alexa Fluor^®^ 594 (red), and DAPI (nuclei; blue) under a confocal microscope. Scale bar = 100 μm. **b** Cx43 and N-cadherin expression in MSCs was analyzed at 4 and 7 days of coculture by WB and depicted as a relative ratio to the respective loading control α-tubulin normalized to the monoculture value on day 4. The results represent three independent experiments.

Direct cell-cell communication via gap junctions is an important mechanism by which bone cells coordinate their activities. Gap junction alpha-1 protein (GJA1), also known as connexin 43 (Cx43), is the most abundant connexin in all bone cells. Extensive research has established a crucial role for Cx43 and subsequent intercellular channels in differentiation, function, and survival of osteoblasts *in vitro* and *in vivo* ^31,32^. Therefore, we evaluated the Cx43 localization and expression during the osteoblast differentiation of MSCs cocultured with DRG neurons in the microfluidic device. For this, we performed IF and Western blotting (WB) using an anti-Cx43 antibody (Fig. 5a, b). We found an increase of Cx43 levels mainly in the cytosol and perinuclear area of MSCs after 4 and 7 days of coculture compared with control MSCs cultured for the same period of time (Fig. 5a, b). Interestingly, the highest expression of Cx43 was detected on day 4 of coculture (Fig. 5a, b). These observations show that DRG neurons have a positive impact on Cx43 levels in MSCs during osteoblastogenesis.

Several lines of evidence have indicated that N-cadherin, also known as Cadherin-2 or neural cadherin, regulates the osteoblastogenesis^33^. On the light of these findings, we conducted IF and WB against N-cadherin in order to investigate the impact of coculture with DRG neurons on N-cadherin localization and expression of MSCs maintained in OIM (Fig. 5a, b). We observed that MSCs cocultured with DRG neurons for 4 days exhibited a sharp increase of N-cadherin levels compared with MSCs cultured alone mostly in the cytosol and perinuclear region (Fig. 5a, b). Conversely, the levels of N-cadherin after 7 days of coculture were lower than MSCs in monoculture (Fig. 5a, b). In both mono and cocultured MSCs, the staining of N-cadherin was diffuse, with a nonjunctional, as well as junctional localization (Fig. 5a). These findings demonstrate that coculture with DRG neurons mediates N-cadherin expression changes in MSCs during osteoblast differentiation.

Taken together, these findings suggest that SNS modulates the Cx43 and N-cadherin expression in MSCs during osteoblastogenesis.

### DRG neurons promote the activation of the Canonical/β-catenin Wnt signaling pathway in MSCs

**Fig. 6:**
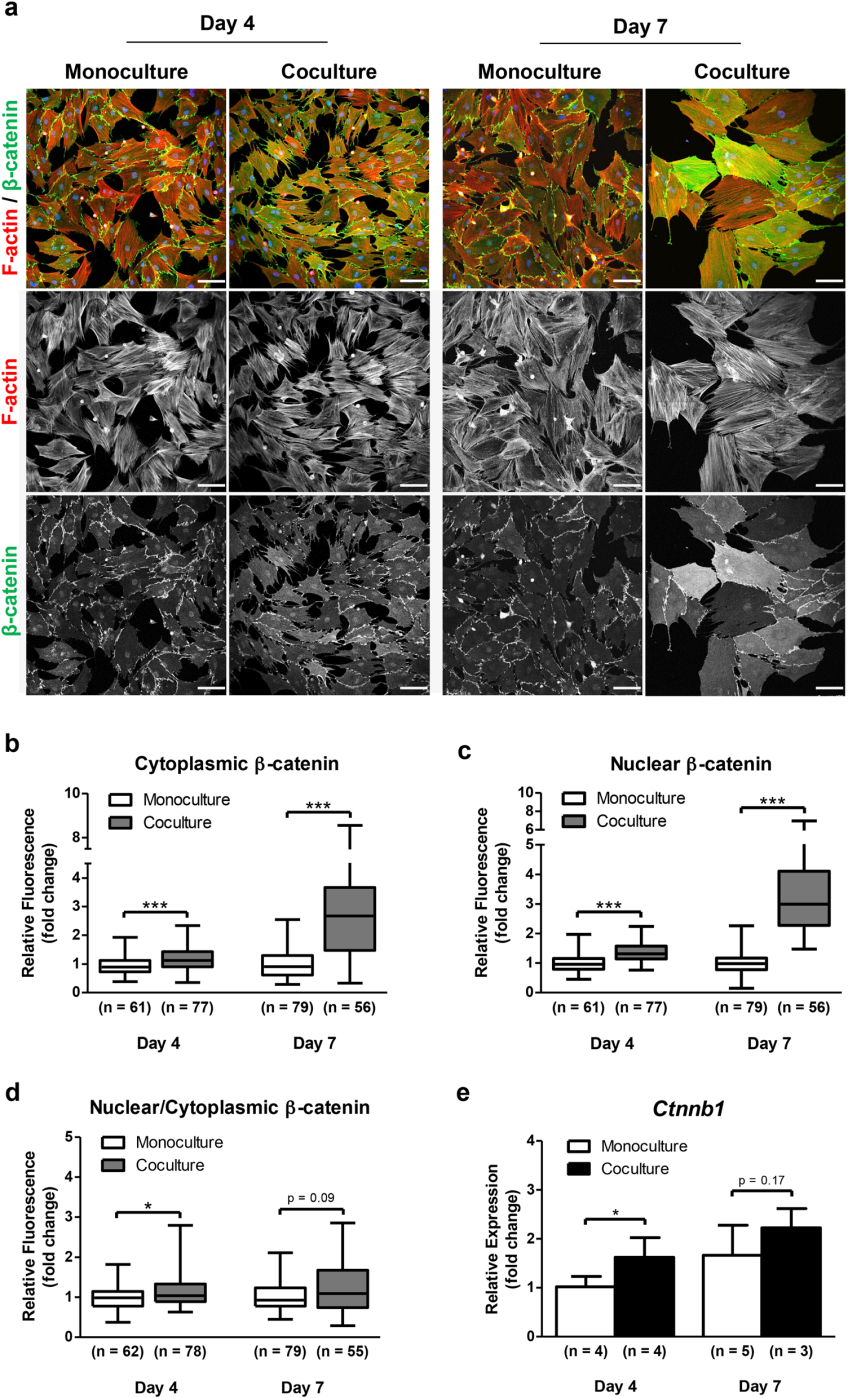
DRG neurons induce cytoplasmic accumulation of β-catenin and its translocation into the nucleus in MSCs Sensory neurons derived from rat DRG (5 × 10^4^ cells/cm^2^) and rat bone marrow MSCs (10^4^ cells/cm^2^) were cocultured in microfluidic devices for 7 days. DRG neurons were maintained in DMEM supplemented with 2% (v/v) B-27 and 1 μM AraC; MSCs were incubated in OIM composed of DMEM-low glucose with 10% (v/v) FBS, 1 × 10^−9^ M dexamethasone, 10 mM β-glycerophosphate, and 50 μg/mL ascorbic acid. **a** Subcellular distribution of β-catenin in MSCs was evaluated at 4 and 7 days of coculture by IF using antibodies directed against β-catenin coupled to Alexa Fluor^®^ 488 (green), and DAPI (nuclei; blue) under a confocal microscope. Scale bar = 100 μm. **b–d** The level of fluorescence for β-catenin was measured at 4 and 7 days of coculture by using the ImageJ software in each MSC and normalized to the monoculture value on day 4. Data expressed as median, 25 and 75 percentiles, min/max. (*n*) indicates the total number of cells counted for each group. **e** Expression profile of *Ctnnb1* in MSCs was assessed at 4 and 7 days of coculture by RT-qPCR and depicted as a relative ratio to the housekeeping gene *Hprt1* normalized to the monoculture value on day 4. Data expressed as mean ± SD. (*n*) indicates the total number of samples for each group. *p < 0.05; ***p < 0.001 statistically different from monoculture. The results represent three independent experiments.

It has been widely demonstrated that the canonical Wnt signaling pathway is a key regulator of osteogenesis by determining the cell fate decision of MSCs. β-catenin is the molecular node of this pathway and its stability and localization are crucial for intracellular signal transduction^34^. To elucidate whether this mechanism is implicated in osteoblast differentiation of MSCs cocultured with DRG neurons in the microdevice, we evaluated the subcellular distribution of β-catenin in MSCs by IF (Fig. 6). In our study, we observed a significant increase of cytoplasmic β-catenin expression in cocultured MSCs for 4 and 7 days compared with the respective control MSCs (Fig. 6a, b). We also found that the nuclei of MSCs in coculture for 4 days exhibited greater β-catenin levels than those in monoculture, indicating more β-catenin translocated from the cytoplasm to the nucleus (Fig. 6a, c, and d). Interestingly, the addition of DRG neurons to the MSCs culture resulted in a 3.4-fold increase of nuclear β-catenin in MSCs on day 7 of coculture, although not associated with a significant transfer of β-catenin into the nucleus (Fig. 6a, c, and d). Furthermore, a significant 1.6-fold increase of β-catenin gene (*Ctnnb1*) was detected in cocultured MSCs for 4 days compared with MSCs in monoculture (Fig. 6e). In contrast, no differences were observed in *Ctnnb1* expression between MSCs in mono and coculture after 7 days (Fig. 6e). These results show that coculture with DRG neurons promotes the cytosolic accumulation and nuclear translocation of β-catenin in MSCs.

**Fig. 7:**
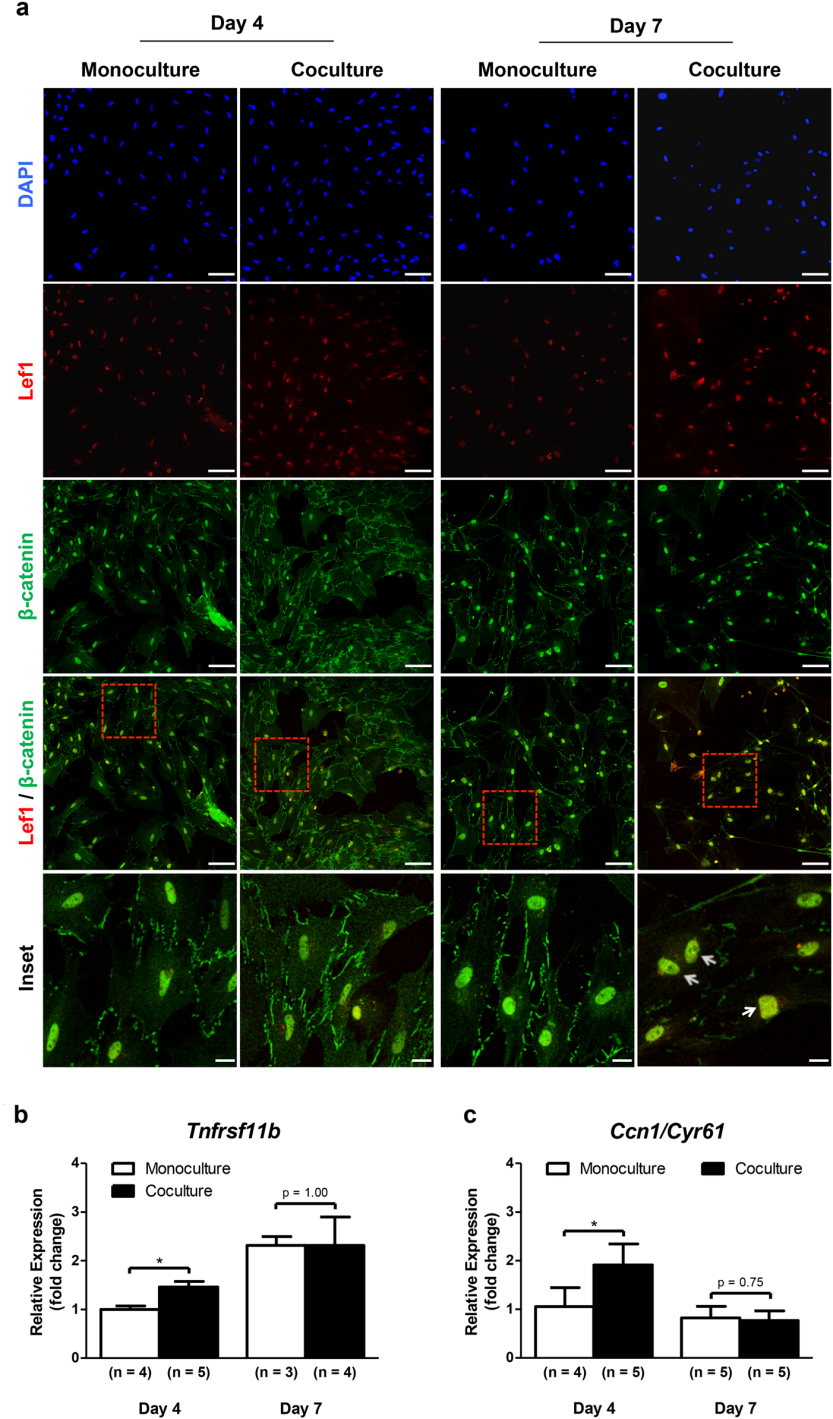
DRG neurons lead to colocalization between β-catenin and Lef1 into the nucleus, where together regulate the expression of Wnt-responsive genes in MSCs Sensory neurons derived from rat DRG (5 × 10^4^ cells/cm^2^) and rat bone marrow MSCs (10^4^ cells/cm^2^) were cocultured in microfluidic devices for 4 and 7 days. DRG neurons were maintained in DMEM supplemented with 2% (v/v) B-27 and 1 μM AraC; MSCs were incubated in OIM composed of DMEM-low glucose with 10% (v/v) FBS, 1 × 10^−9^ M dexamethasone, 10 mM β-glycerophosphate, and 50 μg/mL ascorbic acid. **a** Nuclear colocalization of active-β-catenin and Lef1 in MSCs was evaluated at 4 and 7 days of coculture by IF with antibodies directed against active-β-catenin coupled to Alexa Fluor^®^ 488 (green), Lef1 coupled to Alexa Fluor^®^ 594 (red), and DAPI (nuclei; blue) under a confocal microscope. Arrows point to nuclear colocalization. Scale bar = 100 and 20 μm inset images. **b** and **c** Expression profile of *Tnfrsf11b* and *Ccn1/Cyr61* in MSCs was assessed at 4 and 7 days of coculture by RT-qPCR, and depicted as a relative ratio to the housekeeping gene *Hprt1* normalized to the monoculture value on day 4. Data expressed as mean ± SD. (*n*) indicates the total number of samples for each group. *p < 0.05 statistically different from monoculture. The results represent three independent experiments.

It is well documented that β-catenin exerts its effect on gene transcription by functioning as a transcriptional co-activator. The best-characterized binding partners for β-catenin in the nucleus are the members of the T-cell factor (Tcf)/lymphoid enhancer factor (Lef) DNA-binding family. In osteoblast progenitor cells, this interaction leads to transcriptional activation of Wnt target genes, particularly *Runx2/Cbfa1*, *Sp7*, TNF receptor superfamily member 11b (*Tnfrsf11b*), and CCN family member 1 (*Ccn1*), also known as cysteine-rich angiogenic inducer 61 (*Cyr61*), which regulate the osteoblastogenesis^35–37^. Based on this, we first examined the nuclear colocalization between active-β-catenin and Lef1 in MSCs committed to osteoblast lineage and cocultured with DRG neurons in the microfluidic platform. To achieve this, we performed IF using double labeling against these proteins (Fig. 7a). The yellow fluorescence resulting of the β-catenin and Lef1 channels overlap showed a strong colocalization between these two proteins in nuclei of cocultured MSCs for 7 days (Fig. 7a, arrows). On the other hand, there seemed to be no differences between MSCs in mono and coculture after 4 days (Fig. 7a). Then, we analyzed the expression of *Tnfrsf11b* and *Ccn1/Cyr61* Wnt-responsive genes in MSCs by RT-qPCR after 4 and 7 days of the coculture. We found a significant upregulation of *Tnfrsf11b* (1.5-fold change) and *Ccn1/Cyr61* (1.8-fold change) in MSCs cocultured with DRG neurons for 4 days, when compared with monocultured MSCs (Fig. 7b and c). The same results were not verified for day 7 (Fig. 7b and c). These findings demonstrate that coculture with DRG neurons controls the Lef1-responsive transcriptional activation of *Tnfrsf11b* and *Ccn1/Cyr61* in MSCs during osteoblast differentiation.

Together, these results suggest that SNS regulates the activation of the canonical/β-catenin Wnt signaling pathway in MSCs during osteoblastogenesis.

## Discussion

The majority of the existent *in vitro* studies focusing on the communication between the SNS and bone tissue were performed with coculture models using direct cell-cell contact^14–16^. However, these models have been widely criticized for not mimicking the *i* *n vi* *vo* scenario of bone sensory innervation. To circumvent this drawback, we developed microfluidic devices in order to establish a controlled and isolated coculture system between DRG neurons and MSCs, where only the neurites reach the MSCs as it happens in the in vivo situation. Despite embryonic DRG neuron cultures have the advantage of higher cell density and greater neurite outgrowth they are dependent on neurotrophins to survive. Most neurotrophins, especially nerve growth factor (NGF or beta-NGF), are pleiotropic causing multiple biological effects^38^. For this reason we focused on the use of adult DRG neurons.

Osteoblast differentiation of MSCs *in vitro* can be achieved by the presence of dexamethasone, ascorbic acid and β-glycerol phosphate in the culture medium^39,40^. Under these culture conditions, the commitment/maturation of MSCs towards osteoblast lineage have been divided into three hallmark stages. The first stage is marked by the increase in the number of cells as result of MSCs proliferation. This is followed by cell cycle arrest and early osteoblastic differentiation of MSCs associated with ECM deposition/maturation. The final stage is characterized by the late osteoblastic differentiation related to ECM mineralization^41^. The temporal pattern of expression of genes/proteins characterizing the different stages of osteoblast lineage development varies according to the cell culture models^42^. The formation of bone ECM during the second stage of osteoblastogenesis is associated with the maximal expression of Alp and transcriptional activation of early osteoblastic genes, particularly *Col1a1*. In the later stages of osteoblast differentiation while the Alp levels are decreased, the expression of genes related to mineralization, such as *Bglap* and *Spp1* (encoding osteopontin), are increased at the transcriptional level^41,43^. Accordingly, in our research, we found a significant upregulation of early osteoblast-specific genes, especially *Runx2*, *Sp7*, and *Col1a1* in MSCs cocultured with DRG neurons for 4 days in the presence of OIM. We also observed a peak on day 4 of Alp levels in cocultured MSCs under the same culture conditions. Moreover, after 7 days of coculture under osteogenic stimulation, the mRNA expression of *Bglap* in MSCs was significantly increased, and the levels of Alp tended to decline. These results indicate that on day 4 of coculture with DRG neurons, MSCs were in the second stage of osteoblastogenesis. We can also presume that on day 7 of coculture, the now preosteoblasts had progressed to the final stage to become mature osteoblasts. The effect of DRG neurons on transcriptional regulation of osteoblast-marker genes in MSCs after 14 days of coculture in the presence of OIM was also evaluated, but no differences were detected between mono and cocultured MSCs (data not shown). Interestingly, there were no differences in the transcription of osteoblast-related genes and also in the activity of Alp between MSCs in mono and coculture without OIM (Supplementary Figure S2). Together, these results provide a strong evidence for a role of DRG neurons in enhancing the osteoblast differentiation from MSCs. However, its effect *per se* is not sufficient to induce the osteoblastogenesis *in vitro*.

Multiple studies have provided insights into the importance of Cx43 in osteoblast and bone formation^31,32^. Specifically, it was shown that mutations in *GJA1* impair the osteoblast lineage development and cause skeletal anomalies associated with oculodentodigital dysplasia^44^. The impact of the SNS on Cx43-mediated osteoblastogenesis is unknown, although few reports indicate a role of neuropeptides in this process. Specifically, it was demonstrated that CGRP and Substance P are implicated on the promotion of osteoblast proliferation and activity through an increase of gap junctional intercellular communication (GJIC) and Cx43 expression^45,46^. Recently, an *in vitro* study showed that Cx43 expression and GJIC are increased during the osteoblast formation, both at transcriptional and translational levels. The authors also demonstrated that Cx43 and subsequent GJIC play a central role in early osteoblast differentiation by mediating the expression of Runx2^47^. These reports may explain the sharp increase of Cx43 expression in MSCs cocultured with DRG neurons for 4 days in the presence of the OIM. Despite a significant decrease on day 7, the Cx43 levels in cocultured MSCs were significantly higher than in MSCs cultured alone for the same period. Interestingly, we observed a mainly cytosolic and perinuclear distribution of Cx43. Some studies have proposed that the subcellular localization of Cx43 is related to different signaling activities, which contribute differently to the regulation of cell proliferation and differentiation^48^. These results show for the first time that DRG neurons have a positive effect on MSCs Cx43 expression and maybe on GJIC during osteoblast differentiation, especially at an early stage.

N-cadherin is another functional protein involved in osteogenesis^33^. However, its impact on osteoblast lineage development is not consensual among experts. Many reports have shown that N-cadherin hinders the osteoblast formation and function^49,50^. On the other hand, we and others have demonstrated that N-cadherin is required for early stages of osteoblastogenesis^51,52^. A recent in vivo study described a dual action of this protein on osteolineage cells depending upon their differentiation level. The authors demonstrated that N-cadherin maintains the pool of osteoprogenitor and stem cells and prevents the osteoblast differentiation of lineage-committed cells and function of mature osteoblasts^53^. In our research, we detected an increase of N-cadherin expression in MSCs cocultured with DRG neurons for 4 days, followed by a decrease three days later. These findings indicate that DRG neurons differentially modulate the expression of N-cadherin in MSCs according to their osteoblast developmental stage.

Different mechanisms have been proposed for regulation of osteoblast differentiation by N-cadherin. One of them is the increase of N-cadherin-mediated cell-cell adhesion, resulting in activation of signaling events that promote osteoblast gene expression. Interestingly, was suggested that the increase of cell–cell adhesion leads to an increase of Cx43-mediated GJIC and subsequent activation of osteoblast-marker genes, both at transcriptional and translational levels. It has also been reported that the interaction of N-cadherin with molecular players of the Wnt signaling pathway triggers intracellular signal transduction. In particular, the interaction of N-cadherin with β-catenin at the plasma membrane results in β-catenin sequestration, reduction of the cytosolic β-catenin pool and inhibition of the canonical Wnt signaling. Finally, N-cadherin can interact with the Wnt-co-receptors LRP5 or LRP6, promoting the β-catenin degradation, reduction of Wnt/β-catenin signaling, and decrease of osteoblastogenesis^54^.

In this study, we detected a sharp increase in cytoplasmic accumulation of β-catenin and its translocation into the nucleus of MSCs cocultured with DRG neurons. In addition, the IF staining showed that β-catenin largely colocalized with Lef1 in the nucleus of MSCs cocultured for 7 days. More importantly, we found an upregulation of Wnt target genes implicated in the osteoblast lineage development in MSCs cocultured with DRG neurons for 4 days. These results reveal for the first time that DRG neurons promote the activation of the canonical Wnt signaling pathway in MSCs. Interestingly, recent reports have indicated that this pathway directly increases the metabolism of osteoblast-lineage cells by stimulating the aerobic glycolysis, glutamine catabolism, as well as fatty acid oxidation^55^. Consistently, we observed an increase in the metabolic activity of MSCs cocultured with DRG neurons. However, we cannot exclude that the sensory neurons may also be acting through other signaling pathways. Indeed, a recent study showed that NGF-TrkA signaling by sensory nerves coordinates the osteoprogenitor lineage progression and bone formation in vivo^56^.

**Fig. 8:**
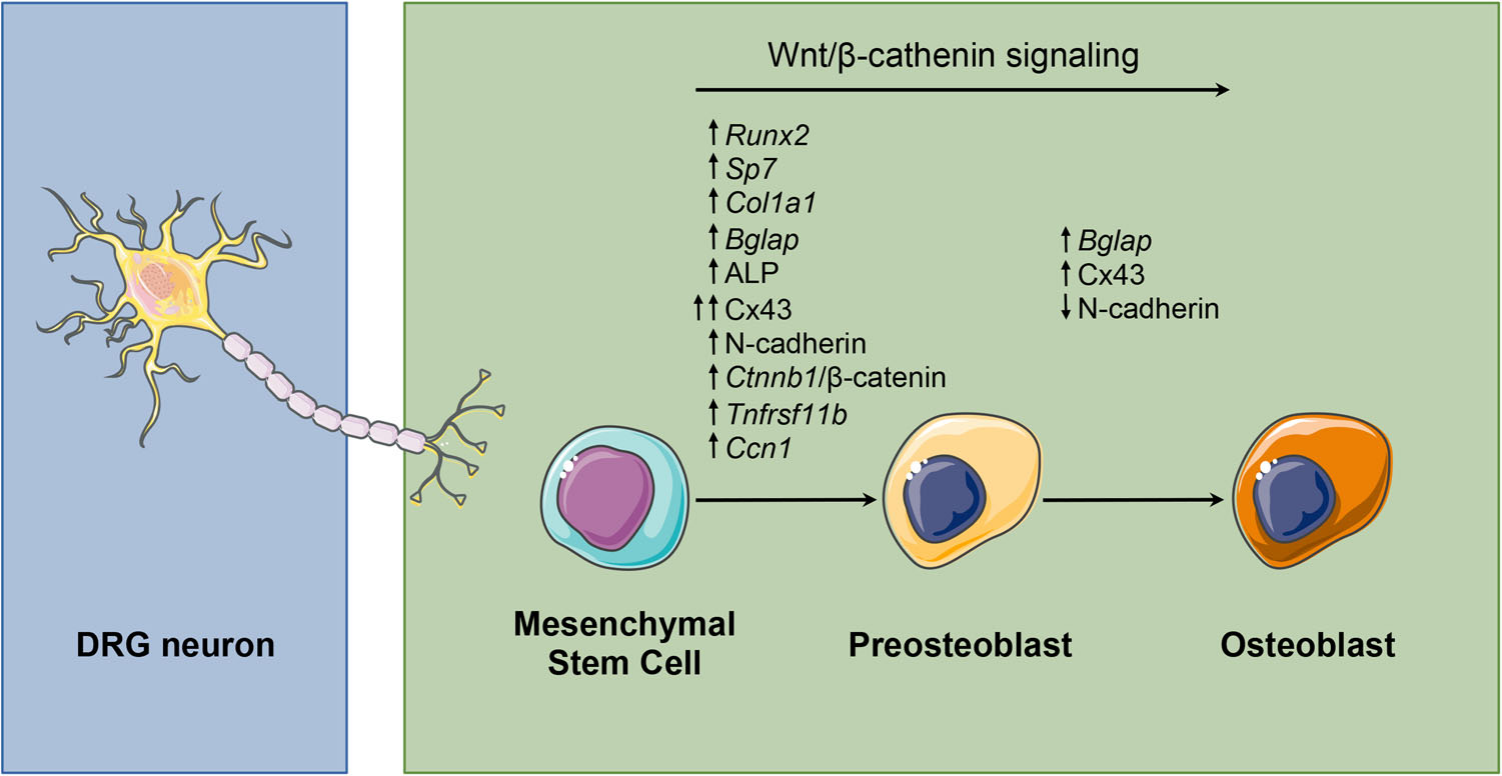
Role of DRG neurons in different phases of osteoblast differentiation from MSCs DRG neurons promote the osteogenic differentiation potential of MSCs by regulating the canonical/β-catenin Wnt signaling pathway and expression of osteoblast-related genes/proteins in MSCs.

The present study provides several lines of evidence that DRG neurons play an incremental role for osteoblastogenesis by acting directly on MSCs and activating the Wnt/β-catenin signaling pathway. Furthermore, we were able to define the temporal pattern of expression of genes/proteins involved in the ability of MSCs to undergo osteogenic differentiation in response to DRG neurons communication (Fig. 8).

Overall, these findings provide new insights into how SNS enhances bone formation and can inform the development of future therapeutic strategies for bone regeneration/repair that takes into account the SNS.

## Materials and methods

### Microfluidic devices fabrication

Microfluidic devices were obtained using standard photolithography and soft lithography procedures^17^.

### Coculture of DRG neurons and MSCs in the microfluidic devices

Microfluidic devices were electrostatically attached over glass coverslips, previously coated with 0.1 mg/mL Poly-D-lysine (PDL, Sigma–Aldrich^®^, St. Louis, MI, USA) and 20 μg/mL laminin (Sigma–Aldrich^®^). After attained the confluence, MSCs were seeded in the axonal side of a microfluidic device at a density of 10^4^ cells/cm^2^ while freshly harvested and dissociated DRG neurons were plated in the somal side at a density of 5 × 10^4^ cells/cm^2^ and left undisturbed in a humidified incubator to allow adhesion. DRG neurons were cultured in growing medium composed of Dulbecco’s modified eagle’s medium (DMEM, Life Technologies™, Gibco^®^, Carlsbad, CA, USA), with 2% (v/v) B-27 Serum-Free Supplement^®^ (B-27, Gibco^®^) and 1% (v/v) penicillin/streptomycin (Pen/Strep, Gibco^®^). MSCs were incubated in OIM, which consisted of DMEM—low glucose supplemented with 10% (v/v) fetal bovine serum (FBS, P30-3305, PAN™—Biotech, Aidenbach, Germany), 1% (v/v) Pen/Strep, 1 × 10^−9^ M dexamethasone (Sigma–Aldrich^®^), 10 mM β-glycerophosphate (Sigma–Aldrich^®^) and 50 μg/mL ascorbic acid (Sigma–Aldrich^®^). Non-neuronal cells were eliminated using 1 μM cytosine arabinofuranoside (AraC, also known as cytarabine, Sigma–Aldrich^®^). Cocultures were maintained for 7 days in a humidified atmosphere, and the media were renewed on day 4 of coculture.

### Immunofluorescence staining

DRG neurons and MSCs were washed with Phosphate-Buffered Saline (PBS; 0.1 M, pH 7.4) and fixed in 1% (v/v) paraformaldehyde (PFA, MM France, Brignais, France) for 10 min at room temperature (RT). After a wash with PBS, cells were permeabilized for 5 min with 0.1% (v/v) triton^®^ X-100 (EDM Millipore, Billerica, MA, USA) and washed once more. Then, cells were blocked with 1% (w/v) bovine serum albumin (BSA, GE Healthcare, Chicago, IL, USA) for 30 min at RT to minimize the non-specific binding. Cells were further incubated with the primary antibody solution for 1 h at RT. After a wash with PBS, cells were incubated for 45 min in the dark with a solution containing the appropriated conjugated secondary antibody. Nuclei were counterstained for 5 min with 4′,6-diamidino-2-phenylindole (DAPI, 1:5000; Life Technologies™, Molecular Probes^®^). All dilutions were made in PBS. Images were acquired in a Leica TCS SPE Confocal Laser Scanning Microscope (Leica, Wetzlar, Germany).

### Cell metabolic activity and proliferation assays

DNA content of MSCs was determined using CyQUANT™ Cell Proliferation Assay Kit (Life technologies™, Molecular Probes^®^), according to manufacturer’s instructions.

The resazurin-based assay was performed for detection of metabolic activity of MSCs. Briefly, 400 μL of fresh culture medium supplemented with 0.01 mg/mL (w/v) resazurin (Sigma–Aldrich^®^) was added directly to each microfluidic device. MSCs were incubated at 37 °C for 4 h, and then 100 μL from each device was transferred to a 96-well microplate. Fluorescence (λem = 530 nm, λex = 590 nm) was measured on a VICTOR™ ×3 Multilabel Plate Reader (PerkinElmer, Waltham, MA, USA). DNA content of MSCs was determined using CyQUANT™ Cell Proliferation Assay kit (Life technologies™, Molecular Probes^®^), according to manufacturer’s instructions.

### RNA extraction, cDNA synthesis, and RT-qPCR analysis

Total RNA was extracted from MSCs by using the RNeasy^®^ Plus Micro Kit (Qiagen, Hilden, Germany) according to the manufacturer’s protocol. RNA final concentration and purity (OD_260/280_) was determined using a NanoPhotometer^®^ P 330 (Implen GmbH, Munich, Germany). Fifty nanogram of total RNA was reverse transcribed into cDNA using the Maxima Reverse Transcriptase kit (Thermo Scientific™, Thermo Fisher Scientific, Waltham, MA, USA), according to the manufacturer’s protocol. RT-qPCR experiments were run using a CFX Connect™ Real-Time PCR Detection System (Bio-Rad Laboratories, Hercules, CA, USA) and analyzed with the CFX Manager™ software, version 3.0 (Bio-Rad Laboratories). Target gene expression was quantified using the cycle threshold (Ct) values and relative mRNA expression levels were calculated as follows: 2^ (Ct reference gene—Ct target gene). Rat ribosomal protein lateral stalk subunit P0 (*Rplp*0), glyceraldehyde 3-phosphate dehydrogenase (*Gapdh*) and hypoxanthine phosphoribosyl transferase 1 (*Hprt1*) were used as reference genes.

### Alkaline phosphatase activity detection

Quantitative determinations of Alp were performed in MSCs lysates by using the LabAssay™ ALP kit (Wako Pure Chemical Industries, Ltd., Chūō-ku, Osaka, Japan) following manufacturer’s instructions. Absorbance at 405 nm was measured on a VICTOR™ ×3 Multilabel Plate Reader. Cell lysates were analyzed for protein content using the Pierce™ BCA Protein Assay Kit (Thermo Scientific™) according to the manufacturer’s protocol, and intracellular Alp levels were normalized for total protein concentration.

For cytochemical staining, MCSs were washed with PBS and fixed in 4% (v/v) PFA for 10 min at RT. After a wash with water, cells were incubated for 30 min in a solution with 0.01% Naphtol AS-MX phosphate (Sigma-Aldrich^®^) and 0.03% Fast Violet B salt (Sigma-Aldrich^®^), at RT in the dark. Finally, cells were washed with water and air-dried. Images were acquired in a Leica MZ10F stereomicroscope (Leica).

### Protein extraction, precipitation, and Western blotting analysis

Cells were lysed on ice for 15 min with lysis buffer containing 1% (v/v) triton^®^ X-100, and 1% (v/v) Nonidet-P40 enriched with a protease and phosphatase inhibitor cocktail (Sigma–Aldrich^®^). To separate proteins from cellular debris, cells lysates were spun down and the supernatant (proteins) were quantified using the Pierce BCA Protein Assay Kit (Thermo Scientific™). Fourty microgram of total protein was precipitated with acetone, denatured in loading buffer, separated by sodium dodecyl sulfate-polyacrylamide gel electrophoresis (SDS-PAGE), on a 10% polyacrylamide gel, and electroblotted to the Hybond™-C Extra nitrocellulose membrane (GE Healthcare Life Sciences, Chicago IL, USA). Membranes were blocked for 1 h with 5% BSA and 0.5% Tween-20 in PBS and immunoblotted overnight at 4 °C with primary antibodies. Horseradish peroxidase (HRP)—conjugated secondary antibodies were used accordingly. Immunodetection was carried out with the Clarity™ Western ECL detection kit reagents (Bio-Rad Laboratories). Membranes were visualized on ImageQuant™ LAS 4000 mini (GE Healthcare Life Sciences) and bands intensity was quantified using the ImageJ software.

### Statistical analysis

GraphPad Prism^®^ software, version 5.0 (GraphPad Software Inc, Sandiego, CA, USA) was used for statistical analysis. Results are presented as a bar with the mean ± standard deviation (mean ± SD) or box-plot with median and min to max whiskers. Significant differences between two independent groups were established using the Mann–Whitney *U* test. Values of *p* < 0.05 were considered statistically significant.

## Electronic supplementary material

Supplementary Information

Supplementary Figure S1

Supplementary Figure S2
