# Supplementary material for: Dorsal root ganglion neurons regulate the transcriptional and translational programs of osteoblast differentiation in a microfluidic platform
Source: Cell Death Dis. 2017 Dec 13;8(12):3209. doi: 10.1038/s41419-017-0034-3 (PMC5870602; doi:10.1038/s41419-017-0034-3)
Supplement: Supplementary file 1 — Supplementary Information [file 41419_2017_34_MOESM1_ESM.pdf]

SUPPLEMENTAL DATA

Supplementary Figure S1. DRG neurons and MSCs rat primary cultures characterization. (A) Sensory neurons, isolated from rat DRG and dissociated using a prolonged collagenase treatment followed by mechanical trituration, were cultured in DMEM containing 2 % (v/v) B-27 supplement and 1 % Pen/Strep. Cell morphology was evaluated by IF 7 days after isolation, using an antibody directed against calcitonin gene-related peptide (CGRP) coupled to Alexa Fluor® 488 (green), and DAPI (nuclei; blue) under a confocal microscope. Scale bar = 100 µm. (B and C) Rat bone marrow MSCs were selected by plastic adherence in the presence of DMEM - low glucose supplemented with 10 % (v/v) FBS and 1 % (v/v) Pen/Strep. (B) 5 days after isolation, cell morphology was observed under an inverted phase contrast microscope and (C) immunophenotypic analysis was performed by flow cytometry (CD29, CD90 and CD105 as MSCs specific markers and CD34 and CD45 as hematopoietic markers).

Supplementary Figure S2. DRG neurons do not have the capacity to induce osteoblast differentiation of MSCs in the absence of an osteogenic stimulus. Sensory neurons derived from rat DRG (5 x 10^4 cells/cm^2) and rat bone marrow MSCs (10^4 cells/cm^2) were cocultured in microfluidic devices for 7 days. DRG neurons were maintained in DMEM supplemented with 2 % (v/v) B-27 and 1 µM AraC; MSCs were incubated in standard culture medium composed of DMEM-low glucose with 10 % (v/v) FBS. (A-D and F) Expression profile of *Runx2*, *Sp7*, *Col1a1*, *Bglap,* and *Ctnnb1* in MSCs was assessed at 4 and 7 days of coculture by RT-qPCR and normalized to the monoculture levels on day 4. Gene expression levels were calculated as a relative ratio

to the average value of housekeeping gene *Rplp0*. Data expressed as mean ± SD. (n) indicates the total number of samples for each group. (E) Alp activity in MSCs was analyzed at 4 and 7 days of coculture by Alp cytochemical staining. The results represent four independent experiments.

SUPPLEMENTAL EXPERIMENTAL PROCEDURES

Microfluidic devices fabrication

Microfluidic devices were obtained using standard photolithography and soft lithography procedures. The first step comprised the fabrication of a master mold, which consists of two layers of photoresist structures on a flat silicon wafer substrate. Afterward, poly (dimethylsiloxane) (PDMS, Sylgard 184, Dow Corning, Midland, MI, USA) was mixed with a curing agent at a w/w of 10:1 and poured onto the silicon wafer. Master mold with PDMS was then placed in a vacuum desiccator for 15 min to remove air bubbles from the PDMS. Subsequently, PDMS was cured at 60 ºC for 2 h, and microfluidic chambers were cut and separated from the master mold. Reservoirs were punched out using an 8 mm tissue biopsy punch. Finally, the microfluidic devices were sterilized in 70 % (v/v) ethanol and allowed to dry in a laminar flow hood.

Before use, microfluidic devices were irradiated with UV light for 20 min to create reactive species on the surface, which when placed together with glass coverslips to form an electrostatic bond. UV irradiation also makes the surface hydrophilic helping the addition of liquids.

Rat bone marrow mesenchymal stem cells isolation

Primary bone marrow MSCs were obtained from a healthy male 6-10 week-old Wistar rat. Briefly, after sacrificing the rat by CO_2, back limbs were harvested and soft tissue attached to the skeleton was removed. Femora and tibia were washing with ice-cold hank's balanced salt solution (HBSS, Gibco®), supplemented with 10 % (v/v) Pen/Strep, and the extremities were clipped to expose the marrow. Bones were then transferred to a 1.5 mL microcentrifuge tube, which was subsequently inserted into a 15 mL centrifuge tube and centrifuged for 1 minute at 3000 rpm to collect the marrow. The obtained pellet was resuspended in standard culture medium, which consisted of DMEM - low glucose with 10 % (v/v) FBS and 1 % (v/v) Pen/Strep. Cells were dispersed using a 21G needle for 4-6 times and filtered through a 100 µm cell strainer to remove bone fragments and cell clumps. The cell suspension was then centrifuged at 1200 rpm for 5 min, resuspended in standard culture medium, and maintained in a humidified incubator (37 °C and 5 % CO_2). The medium was changed after 3 days to remove non-adherent cells, and subsequently, the adherent cells were cultured to confluence (the medium was renewed every 3/4 days). Cells from the first passage were used in all studies.

Rat dorsal root ganglion neurons isolation

Primary DRG neurons were obtained from healthy male 6-10 week-old Wistar rats. Briefly, after sacrificing the rats by CO_2, spinal columns were removed and placed in HBSS with 10 % (v/v) Pen/Strep. Columns were opened from the caudal to the rostral end with scissors to reveal the DRG. Then, DRG were individually recovered, placed in DMEM and the nerve trunks removed with a scalpel blade. Subsequently, DRG were digested with 10 mg/mL Collagenase, Type IV (Gibco®) for 2 h at 37 ºC. After

centrifugation at 1200 rpm for 5 min, the pellet was resuspended in DMEM. Afterward, DRG were mechanically dissociated using fire-polished glass Pasteur pipettes (full diameter and ½ diameter). The cell suspension was then centrifuged at 1200 rpm for 5 min 3 times and resuspended in growing medium composed of DMEM with 2 % (v/v) B-27, 1 µM cytosine arabinofuranoside (AraC, also known as cytarabine), and 1 % (v/v) Pen/Strep.

Flow cytometric analysis

Trypsinized MSCs (5 x 10^4 cells) were fixed in 1 % (v/v) PFA for 10 min at 4 °C. Cells were then washed with PBS and incubated in the dark with antibodies anti-CD29-Cy5, anti-CD34-FITC, anti-CD105-Cy5 (20 µg/mL; Bioss Antibodies Inc., Woburn, MA, USA), anti-CD45-PE-CY5, and anti-CD90-FITC (20 µg/mL; Becton Dickinson, East Rutherford, NJ, USA) for 60 min at 4 °C. Matched isotype control antibodies were used as negative controls. Cells were then washed twice with PBS and resuspended in PBS. For immunophenotyping analysis, a typical forward and side scatter gate was set to exclude dead cells and aggregates. A total of 7000 events in the gate were collected and fluorescence was analyzed with a BD Accuri™ C6 Flow Cytometer (Becton Dickinson, Franklin Lakes, NJ, USA) using the CFlow® Plus software.

Pairs of primers used for RT-qPCR analysis.

Target	Forward Primer (5′- > 3′)	Reverse Primer (5′- > 3′)	Predicted Length (base pairs)
Bglap	GAGGACCCTCTCTCTGCTCA	GGTAGCGCCGGAGTCTATTC	178
Ccn1	TCAAGTGTGACCCGGACTG	GACCAGCTTCTTCCTCCACTT	192
Col1a1	GACTGTCCCAACCCCCAAAA	CTTGGGTCCCTCGACTCCTA	102
Ctnnb1	GAAAATGCTTGGGTCGCCAG	CGCACTGCCATTTTAGCTCC	99
Gapdh	GGCATTGCTCTCAATGACAA	TGTGAGGGAGATGCTCAGTG	223
Hprt1	AGCCTAAAAGACAGCGGCAA	GGCCACAGGACTAGAACGTC	87
Rplp0	CACTGGCTGAAAAGGTCAAGG	GTGTGAGGGGCTTAGTCGAA	187
Runx2	CCTTCCCTCCGAGACCCTAA	ATGGCTGCTCCCTTCTGAAC	90
Sp7	TGCTTGAGGAAGAAGCTCACTA	GGGGCTGAAAGGTCAGTGTA	148
Tnfrsf11b	GAGTGTAGAGAGGATAAAACGGAG	ACAGAGGTCAATGTCTTGGATG	127

All primers used were designed to detect all transcript variants and to span exon-exon junction in order to ensure that only properly spliced mRNA and not genomic DNA contaminants was amplified. In addition, the RNA extraction was made with a separating step of RNA and DNA.

Antibodies and dilutions used for immunofluorescence (IF) and Western Blotting (WB).

Target	Catalog	Manufacturer	IF	WB
α-Tubulin	T5168	Sigma-Aldrich®, St. Louis, MI, USA	-	1:5000
Active-β-catenin	05-665	EDM Millipore, Billerica, MA, USA	1:100	-
β-III Tubulin	ab18207	abcam, Cambridge, UK	1:100	-
β-catenin	9587S	Cell Signaling Technology, Danvers, MA, USA	1:100	-
CGRP	ab36001	abcam, Cambridge, UK	1:100	-
Cx43	MAB3068	EDM Millipore, Billerica, MA, USA	1:100	1:1000
Lef1	ab37872	abcam, Cambridge, UK	1:100	-
N-cadherin	sc-59987	Santa Cruz Biotechnology, Santa Cruz, CA, USA	1:100	1:500
