# Supplementary figures and images for: Dorsal root ganglion neurons regulate the transcriptional and translational programs of osteoblast differentiation in a microfluidic platform
Source: Cell Death Dis. 2017 Dec 13;8(12):3209. doi: 10.1038/s41419-017-0034-3 (PMC5870602; doi:10.1038/s41419-017-0034-3)

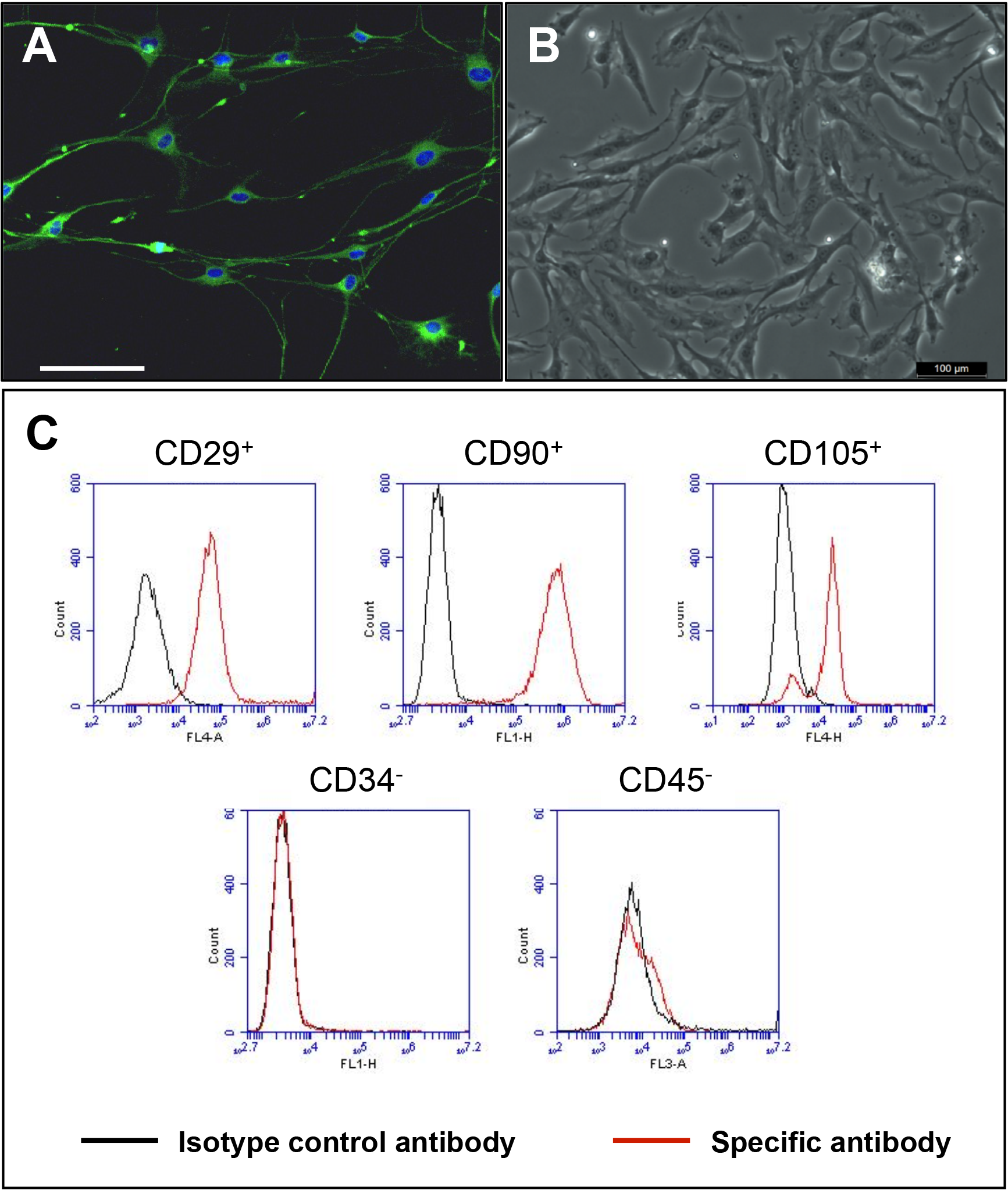

Supplement: Supplementary file 2 — Supplementary Figure S1 [file 41419_2017_34_MOESM2_ESM.tif]

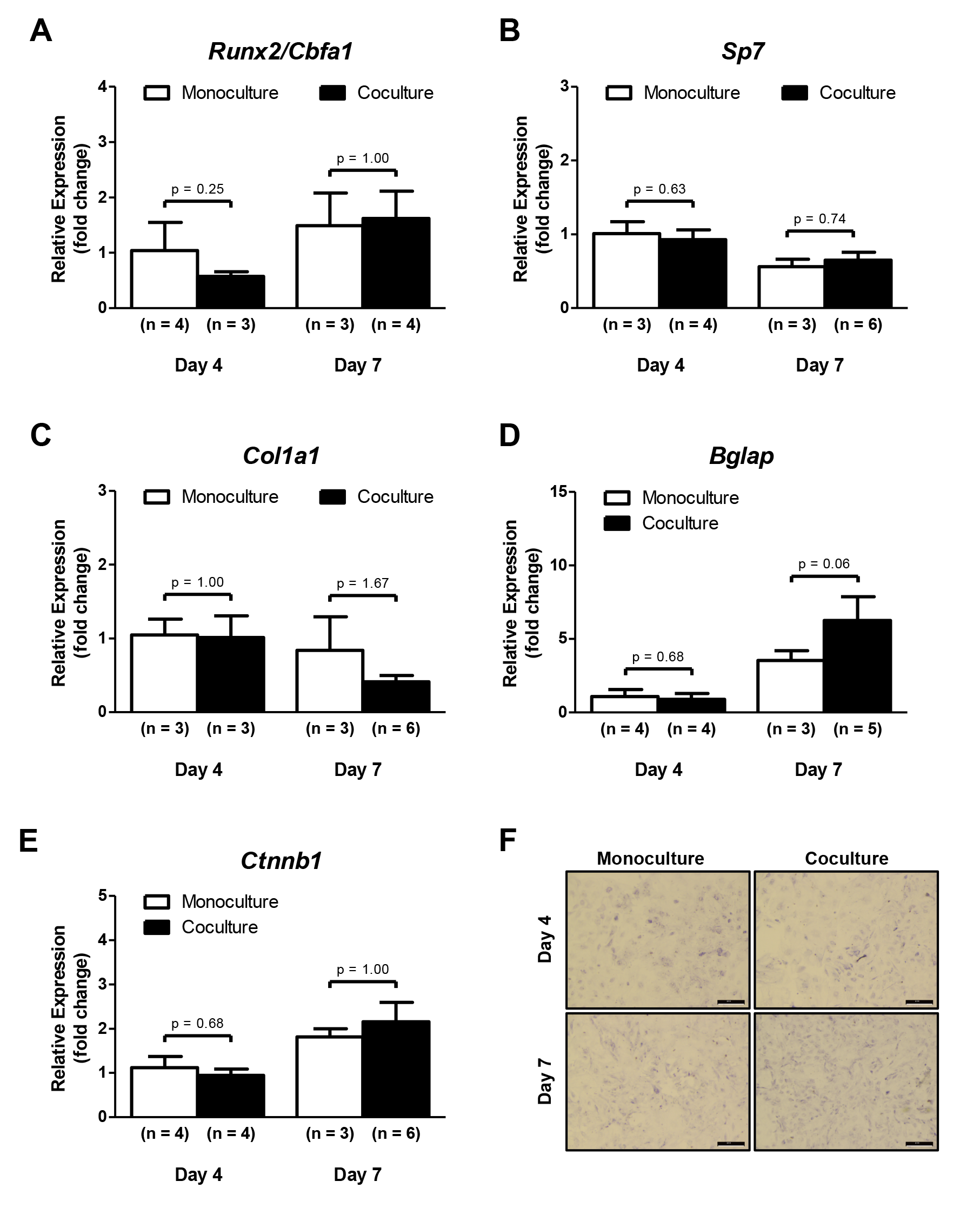

Supplement: Supplementary file 3 — Supplementary Figure S2 [file 41419_2017_34_MOESM3_ESM.tif]
